# Hydroxysteroid 11-Beta Dehydrogenase 1 Overexpression with Copy-Number Gain and Missense Mutations in Primary Gastrointestinal Stromal Tumors

**DOI:** 10.3390/jcm7110408

**Published:** 2018-11-01

**Authors:** Chien-Feng Li, Ting-Ting Liu, Jui-Chu Wang, Shih-Chen Yu, Yen-Yang Chen, Fu-Min Fang, Wan-Shan Li, Hsuan-Ying Huang

**Affiliations:** 1 Department of Pathology, Chi-Mei Medical Center, Tainan 710, Taiwan; angelo.p@yahoo.com.tw; 2 National Institute of Cancer Research, National Health Research Institutes, Tainan 704, Taiwan; 3 Department of Biotechnology, Southern Taiwan University of Science and Technology, Tainan 710, Taiwan; 4 Bone and Soft Tissue Study Group, Taiwan Society of Pathology, Kaohsiung 833, Taiwan; wanshan0129@gmail.com; 5 Department of Pathology, Kaohsiung Chang Gung Memorial Hospital and Chang Gung University College of Medicine, Kaohsiung 833, Taiwan; liutt107@cgmh.org.tw (T.-T.L.); ruizhu0220@gmail.com (J.-C.W.); yu5250@cgmh.org.tw (S.-C.Y.); 6 Division of Oncology, Department of Internal Medicine, Kaohsiung Chang Gung Memorial Hospital and Chang Gung University College of Medicine, Kaohsiung 833, Taiwan; chenyy@cgmh.org.tw; 7 Department of Radiation Oncology, Kaohsiung Chang Gung Memorial Hospital and Chang Gung University College of Medicine, Kaohsiung 833, Taiwan; fang2569@gmail.com; 8 Department of Pathology, Kaohsiung Medical University Hospital, Kaohsiung 807, Taiwan

**Keywords:** gastrointestinal stromal tumor, metabolism, lipid, transcriptome, HSD11B1, gain, mutation, overexpression

## Abstract

The lipid-metabolizing enzymes remain underexplored in gastrointestinal stromal tumors (GISTs). Through transcriptomic reappraisal, hydroxysteroid 11-beta dehydrogenase-1 (*HSD11B1*) was identified as a top-upregulated, progression-associated gene. To validate the clinical relevance of *HSD11B1*, the informative results of Sanger sequencing (*n* = 58), mRNA quantification by branched-chain DNA in situ hybridization assay (*n* = 70), copy number assay by fluorescent in situ hybridization (*n* = 350), and immunohistochemistry (*n* = 350) were correlated with clincopathological variables, *KIT/PDGFRA/BRAF* genotypes, and disease-free survival (DFS). *HSD11B1* was stably silenced to explore its oncogenic function. *HSD11B1* mRNA varied between high-risk and non-high-risk groups (*p* = 0.009) and positively correlated with HSD11B1 immunoexpression (*r* = 0.783, *p* < 0.001). *HSD11B1* copy-number gain (CNG), including polysomy (5.4%) and amplification (12%), associated with HSD11B1 overexpression (*p* < 0.001). Predominantly involving the homodimer interface-affecting exon 6 or exon 7, missense *HSD11B1* mutations (17.2%) were related to high risk (*p* = 0.044), age ≥70 years (*p* = 0.007), and shorter DFS among relapsed cases (*p* = 0.033). CNG was related to unfavorable *KIT/PDGFRA/BRAF* genotypes (*p* = 0.015), while HSD11B1 overexpression was preferential in non-gastric cases (*p* < 0.001). Both abnormalities strongly associated with risk levels (both *p* < 0.001) and shorter univariate DFS (both *p* < 0.0001), and *HSD11B1* CNG remained prognostically independent (*p* < 0.001) with a 3-fold increased hazard ratio. In vitro, *HSD11B1* knockdown significantly inhibited proliferation and caused G2/M arrest. In conclusion, HSD11B1 overexpression may occur owing to CNG, confer a pro-proliferative function, and predict a worse prognosis in GISTs.

## 1. Introduction

Metabolic reprogramming occurs owing to various genetic, epigenetic, and post-translational aberrations in metabolic enzymes that alter signaling pathways of human cancers [1,2]. Recently, mesenchymal tumors have increasingly been exhibiting pathogenetic associations with loss-of-function deregulation of metabolic enzymes, such as isocitrate dehydrogenase in enchondromas and chondrosarcomas, fumarate hydratase in uterine and cutaneous leiomyomas, and succinate dehydrogenase (SDH) in gastrointestinal stromal tumors (GISTs) [3,4]. Notably, SDH-deficient tumors account for a minor subset of GISTs that manifest distinct biological behavior, frequent association with Carney triad or Carney-Stratakis syndrome, and defy effective prognostication by histological assessment [3,4,5,6,7]. Contrarily, the vast majority of GISTs are prognostically predictable using National Institute of Health (NIH) and National Comprehensive Cancer Network (NCCN) risk schemes [8,9]. However, the role of metabolic deregulation remains underexplored in this predominant GIST group that harbors mutually exclusive *KIT* or *PDGFRA* mutations as the tumorigenic drivers and predictors of response to imatinib treatment [10,11]. Hence, it is desirable to identify and investigate the deregulated metabolism-associated enzymes that might affect the disease progression through the provision of cellular energy and building blocks to sustain the growth advantages [1]. 

Compared to the deregulated metabolism of carbohydrates and amino acids, knowledge is limited regarding the deregulation of lipid metabolism in human neoplasms including GISTs [4,12]. Recently, we characterized fatty acid synthase (FASN) (the best-known oncogenic lipid-anabolic enzyme) in GISTs and highlighted its prognostic relevance, biological function to sustain imatinib resistance, and therapeutic potential of dual blockade of FASN and KIT [13]. Regarding lipid catabolic enzymes, we reported the amplification-driven overexpression of phospholipase C isoform β4 (PLCB4) to predict disease-free survival period through the initial reappraisal of published transcriptomic dataset for genes catalogued into the lipid metabolic bioprocess group [14]. Using this focused data-mining approach, we noted that hydroxysteroid 11-beta dehydrogenase 1 (*HSD11B1*) represented another top-rated and differentially upregulated gene associated with high-risk level and development of metastasis in GISTs. Therefore, further genetic, transcriptional, translational, and functional characterization of *HSD11B1* was performed to validate its relevance. *HSD11B1* encodes a microsomal enzyme named 11β hydroxysteroid dehydrogenase isoform 1 and is located on chromosome 1q32.2 [15,16]. In a nicotinamide adenine dinucleotide phosphate (NADP)/NADPH ratio-dependent manner, HSD11B1 bidirectionally catalyzes the interconversion between active cortisol and inactive cortisone through its dehydrogenase and oxidoreductase activities, respectively [15,16]. This biochemical mechanism regulates the availability of local glucocorticoid within the hepatic, adipose, and muscular tissues [15,16,17]. 

In this study, we provided compelling evidence that HSD11B1 immunoexpression level exhibited strong association with DNA copy-number gain (CNG) and mRNA abundance. These genetic and protein expression alterations caused strong adverse effects on the clinicopathological factors and worse outcomes. *HSD11B1* CNG through polysomy or amplification might drive HSD11B1 overexpression in an aggressive GIST subset. Somatic non-synonymous missense *HSD11B1* mutations were detected in 17.2% of GISTs using sequencing and significantly associated with NCCN-defined high-risk, old age, and early recurrences among the relapsed cases. In vitro, we demonstrated the pro-proliferative oncogenic attribute of HSD11B1 in two GIST cell line models using stable RNA interference-mediated silencing. Therefore, our results substantiate the role of HSD11B1 as a novel deregulated lipid-metabolizing enzyme that promotes GIST progression. 

## 2. Materials and Methods 

### 2.1. Reappraisal of Published Transcriptomic Datasets

Transcriptomic datasets of imatinib-naïve GISTs with varying risk levels in Gene expression Omnibus (GSE8167) were reappraised using a previously published method to analyze the probe sets associated with the lipid metabolic bioprocess in Gene Ontology (GO: 0006629) [14]. Unsupervised comparative analysis was performed to identify genes that concordantly exhibited differential expression between the non-high-risk and high-risk cases as well as GISTs with and without metastatic tumors. The fold changes (≥0.2 fold in the log_2_-transformed ratio) in expression and the strength of statistical significance (*p* < 0.01 by Student-*t* test) were considered to rank priority during the selection of candidate genes for validation.

### 2.2. Validation Cohorts 

This study (102-3911B) was approved by the institutional review board of Chang Gung Hospital. *HSD11B1* mRNA expression level was measured by branch-chain DNA in situ hybridization (ISH) assay using QuantiGene system in formalin-fixed primary GISTs (*n* = 86) and adjacent non-tumoral tissue samples (*n* = 10, as the control). *HSD11B1* mRNA quantification was informative in 70 cases, for which HSD11B1 immunoexpression was assessed on whole tissue sections to correlate between mRNA and protein expression. In a large independent cohort comprising 370 primary GIST samples resected prior to 2009, tissue cores (1.5 mm) in triplicate from each sample were previously assembled into tissue microarrays (TMA) [13,14,18], which were recut to perform *HSD11B1*-specific fluorescent in situ hybridization (FISH) and HSD11B1 immunohistochemistry. Among these, 213 cases were previously determined for mutations in *KIT*, *PDGFR,* and v-raf murine sarcoma viral oncogene homolog B (*BRAF*) [13,14], while 58 cases at various risk levels were subjected to *HSD11B1* sequencing in this study. All the cases were imatinib-naïve prior to the tumor relapse. The clinicopathological characteristics of GISTs in TMA-based analyses and *HSD11B1* sequencing were tabulated in Table 1 and Supplementary Table S1, respectively. The details of GISTs used in *HSD11B1* mRNA quantification were previously described [14,18]. 

### 2.3. ISH Assay of Branch-Chain DNA Using QuantiGene System 

A sandwich nucleic acid hybridization method was applied to quantitate the mRNA abundance of housekeeping and target transcripts in the tissue homogenates of formalin-fixed specimens, following the previous protocols [14,18,19]. Specific probes that target *HSD11B1* transcript were customized to detect its expression by using QuantiGene Multiplex 2.0 assay system (Affymetrix/Panomics, Santa Clara, CA, USA). The dioxetane alkaline phosphatase substrate Lumiphos Plus was used to measure intensity using Luminex 100 microplate luminometer (Luminex, Austin, TX, USA). The readout of *HSD11B1* mRNA abundance was determined after normalization using the housekeeping *GAPDH* transcript.

### 2.4. HSD11B1 Locus-Specific FISH 

A bacterial artificial chromosome probe (2312M3, Thermo Fisher, Waltham, MA, USA) spanning *HSD11B1* at 1q32.2 was labeled with spectrum orange. The chromosome 1 control probe that targets the centromeric region (#CHR01-10-GR, Empire Genomics, Buffalo, NY, USA) was labeled with green 5-fluorescein dUTP by following the described method [14,20]. *HSD11B1* copy-number was analyzed on 4-μm TMA sections using these probes and a routine FISH protocol. The average numbers of red and green signals were determined by examining 200 tumor cells for each specimen. Amplification was defined as a ratio of the gene probe signal to the control (i.e., red/green) that exceeds 2.5. Polysomy was identified when the average number of green signals per nucleus was ≥3, with the red/green ratio being ≥1 and <2.5. 

### 2.5. HSD11B1 Immunohistochemistry 

The whole block and TMA sections were microwave-heated to retrieve tissue antigen before incubation with the primary antibody against HSD11B1 (Clone EPR9407(2), 1:50, Abcam, Bristol, UK), followed by detection using ChemMate EnVision kit (K5027, DAKO, Kyoto, Japan). Two pathologists (T.-T.L, W.-S.L) blinded to molecular and survival data independently assessed HSD11B cytoplasmic expression using the previously applied H-score method [14]. Contradictory cases were reviewed by the senior author to obtain consensus. Regarding the TMA cohort, HSD11B1 immunoexpression level was dichotomized into groups with high and low expressions. The cutoff was defined as the median value of individual averaged triplicate H-scores of 350 GISTs with informative data. Among these GISTs, 22 cases harbored wild-type *KIT*, *PDGFRA,* and *BRAF* and exhibited no deficiency in SDHs, as we previously reported [13,14]. 

### 2.6. Mutation Analysis of KIT, PDGFRA, BRAF, and HSD11B1 

The genotyping methods to detect the *KIT* and *PDGFRA* mutations in 213 GISTs were previously reported [20,21,22]. Given the extremely rare concurrence of *BRAF* mutation with either *KIT* or *PDGFRA* mutation [23], 22 GISTs with wild-type *KIT* and *PDGFRA* genes were sequenced for *BRAF* exon-15 that encompasses the mutation hotspot at Val600 [13,14]. 

High-risk (*n* = 20) and non-high-risk (*n* = 38) GISTs (Supplementary Table S1) were selected to explore the potential *HSD11B1* mutations by targeting exon-2, -3, -4, -6, and -7 using ABI3100 sequencer. These represent the coding exons with recurrent mutations that occur in other cancer types according to the catalogue of somatic mutations in cancer (COSMIC) database [24]. The *HSD11B1* exon-1 encodes 5′-untranslated region, while exon-5 is not reported as a recurrently mutated exon in the COSMIC database. Therefore, both these exons were exempted during sequencing. The DNA extraction method, sequences of primer pairs, and PCR thermal conditions of *HSD11B1* sequencing are provided in Supplementary Table S2.

### 2.7. Cell Culture

GIST48 and GIST430 cell lines primarily harbor a homozygous V560D mutation and a heterozygous in-frame deletion in *KIT* exon-11, respectively. After imatinib therapy, GIST48 and GIST430 cells acquired a heterozygous D820A mutation in *KIT* exon-17 and heterozygous missense mutation in *KIT* exon-13, respectively [13,14]. Both cell lines were maintained in Iscove’s modified Dulbecco’s media (Invitrogen, Carlsbad, CA, USA) as previously reported [13,14] and confirmed to exhibit wild-type *HSD11B1* using the primer pairs applied in tumor samples.

### 2.8. RNA Interference 

To gain insight into the role of *HSD11B1* in GIST pathobiology, we established stable clones of GIST48 and GIST430 cell lines using the short-hairpin RNAs that target endogenously expressed *HSD11B1*. The pLKO.1-*shLacZ* and pLKO.1-*shHSD11B1* lentiviral vectors were purchased from Taiwan National RNAi Core Facility and transduced into GIST cells using Lipofectamine 2000 (Invitrogen, Carlsbad, CA, USA) as detailed in Supplementary methods. 

### 2.9. In Vitro Characterization of Oncogenic Functions of HSD11B1 

Supplementary methods detail the methodologies employed for functional validation of HSD11B1 expression in GIST pathobiology, including real-time RT-PCR and western blot assays to confirm knockdown efficiency, bromodeoxyuridine (BrdU) assay to determine cell proliferation rates, flow cytometry-based cell cycle kinetic analysis, and cell migration and invasion assays. 

### 2.10. Statistical Analysis 

Mann-Whitney U test was applied to compare the variation in *HSD11B1* mRNA abundance between the normal and GIST tissues; high-risk and non-high-risk groups; the average time to events between mutated and non-mutated cases of *HSD11B1*-sequenced GISTs that developed relapses (*n* = 20) after primary resection. Pearson correlation analysis was used to evaluate the association between the log_10_-transformed mRNA level and protein expression of HSD11B1. For the TMA cohort, we analyzed the associations of *HSD11B1* copy-number and its protein expression with clinicopathological factors using the Chi-square and Wilcoxon rank-sum tests for categorical and continuous variables, respectively. Follow-up data regarding survival periods were available for 350 cases (median period: 49.9 months; range: 1–247 months). The endpoint was disease-free survival (DFS) that was unaffected by imatinib therapy for disseminated tumors. Based on the previously reported prognostic correlations with DFS, 213 GISTs were dichotomously categorized as favorable or unfavorable genotypes [13,14,22]. The favorable genotypes included the (I) *PDGFRA* mutations in exon-12 or -18, (II) 3′ tandem insertion of *KIT* exon-11 with or without point mutation, and (III) single point mutation of *KIT* exon-11. The unfavorable genotypes comprised (I) Ala502-Tyr503 insertion of *KIT* exon-9, (II) wild type *KIT, PDGFRA*, and *BRAF* without loss of SDHA and SDHB, and (III) 5′ deletion of *KIT* exon-11 with or without point mutation. We used log-rank tests to compare univariate prognostic analyses, among which the significant parameters with univariate (*p* < 0.05) analyses were generally introduced in the multivariate analyses including either NCCN or NIH scheme. Tumor size and mitosis were not incorporated in the multivariate comparisons, as they are component factors of risk stratification. Student’s *t*-test was used to analyze the results of qPCR, BrdU, and flow cytometric assays in cell line samples.

## 3. Results

### 3.1. Differential HSD11B1 mRNA Upregulation in Aggressive GISTs

**Figure 1:**
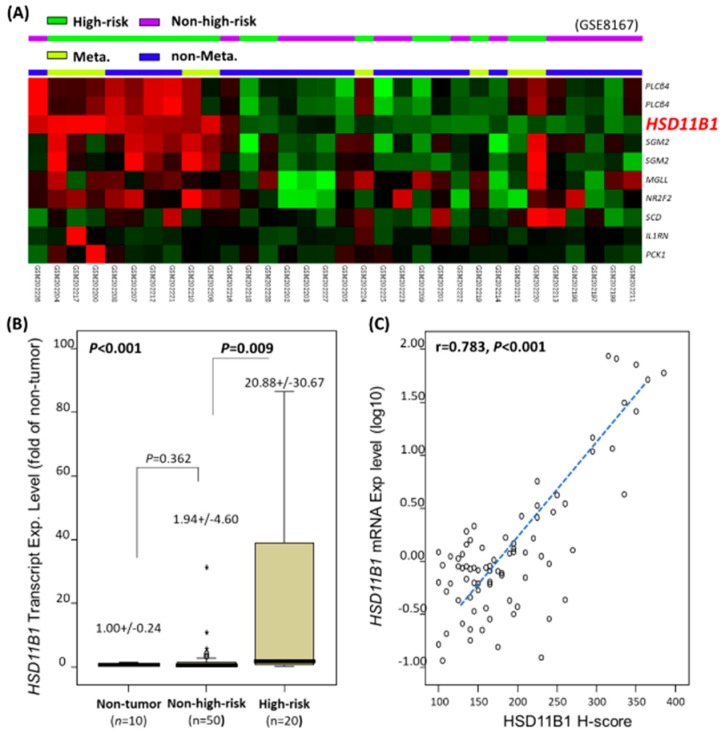
Identification of hydroxysteroid 11-beta dehydrogenase-1 (*HSD11B1*) as one of the top-rated, differentially upregulated lipid metabolism-regulating genes in aggressive gastrointestinal stromal tumors (GISTs) through data mining. (**A**) The results of unsupervised hierarchical clustering analysis of public GIST transcriptome (GSE8167). Profiles of the differentially expressed candidate genes that were segregated into non-high-risk- and high-risk-predominant groups are labeled in purple and green, and non-metastatic- and metastatic-overrepresented groups are labeled as blue and pale green, respectively, on the top of a heatmap. The official names of differentially upregulated (red) and downregulated (green) genes involved in lipid metabolism (GO: 0006629) among which *HSD11B1* was top-rated are presented at right corner; (**B**) Compared to the normal tissues, *HSD11B1* mRNA abundance was differentially upregulated across various risk levels in 70 GISTs (*p* < 0.001) in QuantiGene assay, chiefly attributable to the apparently higher levels in the high-risk group than that in the non-high-risk group (*p* = 0.009); (**C**) In the same set of 70 GISTs used in (B), the scattered plot demonstrated strong correlation between the log_10_-transformed *HSD11B1* mRNA level on *Y*-axis and H-score of HSD11B1 immunoexpression on *X*-axis. Exp., expression; * outliers.

In the reappraisal of GIST transcriptomic dataset (GSE8167), an unsupervised hierarchical clustering was performed for 142 probes that include 77 genes regulating lipid metabolic bioprocess. This clustering analysis enabled the segregation of 32 samples into the non-high-risk- and high-risk-predominant groups, as well as the non-metastatic- and metastatic-overrepresented groups based on the eight differentially expressed genes that were significantly upregulated in the high-risk and metastatic cases (Figure 1A; Supplementary Table S3). In addition to the previously reported *PLCB4* [14], *HSD11B1* represented a top-rank candidate owing to its prominently increased expression fold and strong association with high-risk aggressiveness (*p* < 0.0001; log_2_ ratio = 2.7352) and metastasis (*p* = 0.0001; log_2_ ratio = 2.3984). Therefore, we validated its clinical relevance in two independent tumor cohorts. 

### 3.2. HSD11B1 mRNA Abundance Associated with Risk and Immunoexpression Level 

The *HSD11B1* mRNA abundance readout was informative regarding the 10 normal tissues and 70 primary GISTs, and we were unable to analyze 16 GIST tumors because of RNA degradation. These cases were concordant with their assignment into either high-risk or non-high-risk category by following the NCCN and NIH criteria [8,9]. In 70 informative cases, 49 and 21 were gastric and intestinal tumors, respectively, and classified as high-risk (*n* = 20) and non-high-risk (*n* = 50) cases. The *HSD11B1* mRNA abundance was significantly higher in all the GISTs, compared with that in the normal tissues (*p* < 0.001, Figure 1B) and in high-risk GISTs than that in the non-high-risk group (*p* = 0.009). However, the *HSD11B1* mRNA expression in the non-high-risk group did not significantly vary with respect to that in the reference normal tissues (*p* = 0.360). These findings implied that *HSD11B1* mRNA upregulation was a late event during the evolution of GIST progression. Notably, the log_10_-transformed values of *HSD11B1* mRNA expression were strongly associated with their corresponding immunohistochemical H-scores (*p* < 0.001; *r* = 0.783; Figure 1C), indicating the effective translation of upregulated mRNA into overexpressed protein. 

### 3.3. Association of HSD11B1 CNG and Protein Overexpression with Each Other and Adverse Clinicopathological Factors 

**Figure 2:**
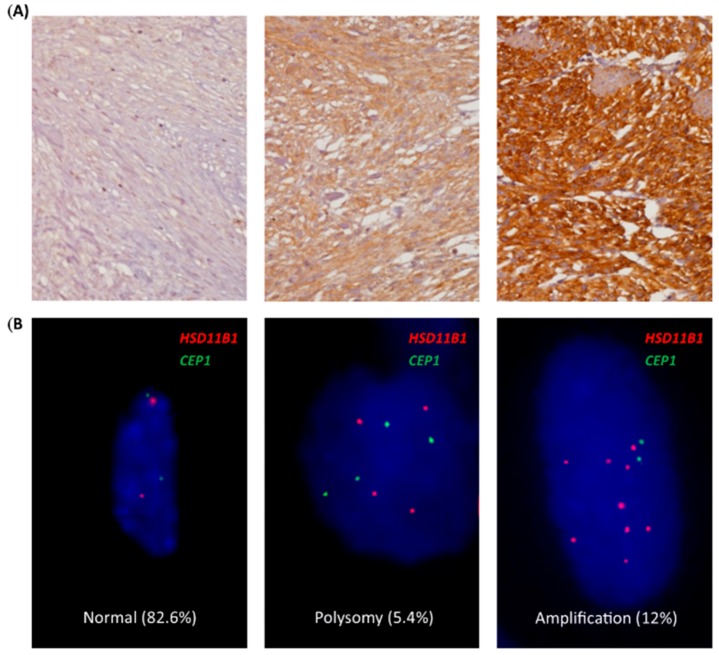
Independent validation to confirm clinical relevance of high hydroxysteroid 11-beta dehydrogenase-1 (HSD11B1) expression and *HSD11B1* copy-number gain (CNG) in tissue microarrays. (**A**) Representative samples of low-risk (left upper), intermediate-risk (middle upper), and high-risk (right upper) gastrointestinal stromal tumors (GISTs) stained with anti-HSD11B1 exhibited no, diffuse moderate, and diffuse strong cytoplasmic reactivity, respectively; (**B**) By using the reference probe labeling the centromeric sequence of chromosome 1 (green), the locus-specific probe that targets *HSD11B1* on 1q32.2 (red) was distinguishable as normal status (left lower; 82.6%), polysomy (middle lower; 5.4%), and amplification (right lower; 12%) in 350 cases by fluorescent in situ hybridization (FISH) assay. CEP 1, chromosome 1 centromere position.

To validate the clinical relevance of *HSD11B1* alterations, the TMA sections from another large cohort were utilized to assess gene copy-number alterations and immunoexpression among which 350 cases were informative for both assays and follow-up data (Table 1 and Table 2). Among these, 88, 100, 65, and 97 were non- or very low-risk, low-risk, moderate-risk, and high-risk cases, respectively, based on NCCN guidelines, while 127, 110, and 113 were very low- or low-risk, intermediate-risk, and high-risk cases, respectively, according to NIH risk scheme. The median of immunohistochemical H-scores of HSD11B1 was 230 (range: 100–365) that was opted to dichotomize the groups exhibiting high and low expressions of HSD11B1 (*n* = 175 each) (Figure 2A). The high expression group was strongly correlated to *HSD11B1* CNG (Figure 2B) detected in 61 (17.4%) cases (*p* < 0.001), including polysomy in 19 (5.4%) and amplification in 42 (12%) cases (Table 1). All the 42 amplified cases and 15 of 19 (79%) polysomic cases exhibited high HSD11B1 expression, while another two-thirds (67.4%, 118/175) of GISTs with overexpressed-HSD11B1 were normal without CNG by performing FISH. These results indicated CNG as an important aberration that drives HSD11B1 expression, while alternative mechanism(s) that upregulate HSD11B1 expression might operate in GISTs. The CNG and increased protein expression of HSD11B1 were strongly associated with the presence of epithelioid histology, increased tumor size, mitosis, and risk level defined by NIH and NCCN schemes (*p* ≤ 0.002 for all the associations, Table 1). *HSD11B1* CNG was significantly more frequent in GISTs that harbored unfavorable *KIT/PDGFRA/BRAF* mutation types (*p* = 0.015), whereas increased HSD11B1 expression (*p* < 0.001) preferentially occurred in non-gastric GISTs. 

### 3.4. HSD11B1 CNG and High Expression Predicted Poor Prognosis in GIST Patients

**Figure 3:**
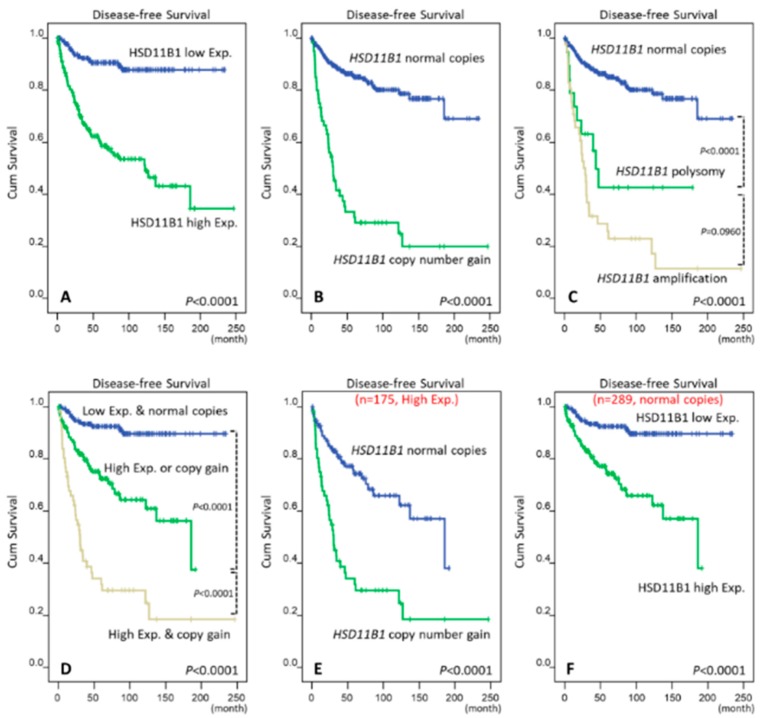
Kaplan-Meier analyses of univariate disease-free survival (DFS) in primary gastrointestinal stromal tumors (GISTs). In all the 350 cases, survival curves were plotted based on (**A**) hydroxysteroid 11-beta dehydrogenase-1 (HSD11B1) immunohistochemical H-scores; (**B**) dichotomy of normal copies versus *HSD11B1* copy-number gain (CNG); and (**C**) trichotomy of normal copies, polysomy, and amplification with remarkable prognostic variation between normal copies and polysomy that exhibits contrast with the comparison between polysomy and amplification; (**D**) Comparison among cases with neither, either or both aberrations of CNG and high HSD11B1 expression; In sub-cohort analyses, *HSD11B1* CNG (**E**) robustly distinguished GISTs with shorter disease-free survival among 175 cases with high HSD11B1 expression, as high HSD11B1 expression; (**F**) did among 289 cases with normal *HSD11B1* gene copies. Exp., expression.

At the univariate level, high HSD11B1 immunoexpression and *HSD11B1* CNG strongly predicted short DFS (Figure 3A,B; Table 2; *p* < 0.0001 in both) in the TMA validation cohort. As the number of *HSD11B1*-polysomic GISTs is relatively low, they were compared regarding their prognostic impact with their counterparts that exhibited normal gene status or amplification. Polysomic cases were found to manifest significantly worse DFS than the cases with normal copy-number (*p* < 0.0001) but trend toward longer DFS (*p* = 0.096) than *HSD11B1*-amplified cases without statistical significance (Figure 3C). Therefore, we justified merging polysomic and amplification cases into a combined CNG group in correlation and prognostic analyses. Interestingly, GIST patients who exhibited *HSD11B1* CNG and high protein expression manifested the worst DFS, followed by patients who harbored either one aberration (*p* < 0.0001) and then by patients with no aberration (*p* < 0.0001). Specifically, the 5-year and 10-year DFS rates in these three prognostically distinct groups (Figure 3D) were 92.5% and 89.5%, 71.0% and 47.1%, and 29.8% and 14.9%, respectively. Even in the subgroup of 175 high HSD11B1-expressing GISTs, the occurrence of *HSD11B1* CNG remarkably shortened the DFS (*p* < 0.0001, Figure 3E) compared with normal gene status. Moreover, high HSD11B1 expression strongly portended worse outcomes in 289 GISTs without *HSD11B1* CNG (*p* < 0.0001, Figure 3F). Collectively, these findings indicated that HSD11B1 overexpression signifies highly aggressive behavior in primary GISTs primarily and secondarily through CNG and unidentified alternative mechanisms, respectively. Additionally, high NCCN-defined risk levels (*p* < 0.0001) as well as unfavorable genotypes (*p* = 0.0005) predicted short DFS (Table 2). 

Regarding the risk levels in multivariate analysis, we separately analyzed the independent prognostic effect of either NCCN or NIH scheme in two distinct models. When NCCN guidelines were incorporated (Table 2), *HSD11B1* CNG (*p* < 0.001, hazard ratio: 3.124) remained prognostically independent along with high NCCN risk levels (*p* < 0.001) and epithelioid histology (*p* = 0.013). However, the factors including high HSD11B1 immunoexpression, unfavorable genotypes, and non-gastric location lost prognostic significance. When NIH scheme was opted (Supplementary Table S4), this multivariate model identified the same three independent prognostic factors of short DFS as by the NCCN scheme-incorporating model, namely, *HSD11B1* CNG (*p* < 0.001, hazard ratio: 3.127), high NIH risk levels (*p* < 0.001), and epithelioid histology (*p* = 0.031). Besides, non-gastric location (*p* = 0.081) and high HSD11B1 immunoexpression (*p* = 0.095) exhibited improved trend toward marginal significance.

### 3.5. Mutation Analysis of HSD11B1 

**Figure 4:**
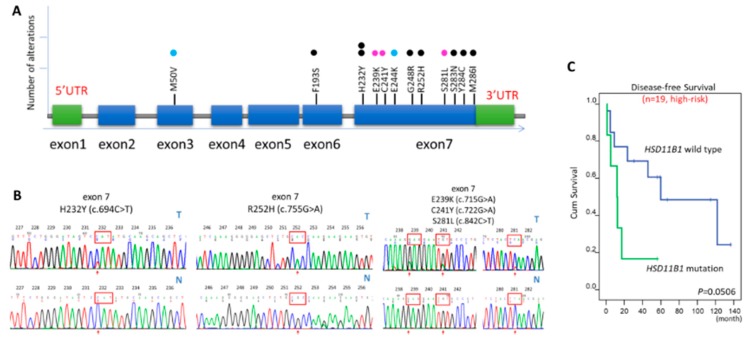
Sanger sequencing analysis to detect hydroxysteroid 11-beta dehydrogenase-1 (*HSD11B1*) mutations. (**A**) A linear diagram that summarizes *HSD11B1* mutation types detected in 10 gastrointestinal stromal tumors (GISTs). Among these, a case with triple mutations in the exon-7, another case with double mutations in the exon-3 and -7, and the remaining eight cases with a single mutation were denoted using pink, blue, and black dots, respectively. Among the eight cases with a single mutation, seven cases predominantly exhibited *HSD11B1* mutations that clustered in exon-7, while one case exceptionally harbored a single mutation (p.F193S) in exon-6; (**B**) Compared with the corresponding adjacent normal tissues, representative sequencing chromatograms of *HSD11B1* exon-7 exhibited the following mutations: (I) a recurrent p.H232Y (c.694C > T) mutation in two cases, (II) an isolated p.R252H (c.755G > A) in one case, and (III) triple mutations of p.E239K (c.715G > A), p.C241Y (c.722G > A), and p.S281L (c.842C > T) in one case. The sequencing chromatograms of the remaining six cases are depicted in Supplementary Figure S1; (**C**) Kaplan-Meier analyses of univariate disease-free survival (DFS) revealed that non-synonymous missense *HSD11B1* mutations exhibited a trend that predicted worse outcomes among the 19 high-risk GISTs that were sequenced in this study.

Sanger sequencing of *HSD11B1* yielded information regarding 58 GISTs. Among these, 10 cases (17.2%) exhibited missense mutations including 6 of 19 high-risk and 4 of 39 non-high-risk tumors. Compared with the corresponding non-neoplastic tissues of eight mutated cases, all the *HSD11B1* mutations were confirmed to exhibit somatic origin (Figure 4A,B; Supplementary Figure S1, Supplementary Table S1). Intriguingly, at least one missense mutation was detected in the exon-7 in 9 of 10 *HSD11B1*-mutated GISTs, including one case with an additional exon-3 mutation (p.M50V) and another with triple exon-7 mutations (p.E239K, p.C241Y, and p.S281L). Two GISTs exhibited the p.H232Y mutation in the exon-7, while the single case without a mutation in the exon-7 harbored a single missense (F193S) mutation in the exon-6. Collectively, these mutations primarily occurred in the last two coding exons (exon-6 and -7) that are presumed to affect the moiety involving the HSD11B1 homodimer interface. The rates of *HSD11B1* mutations were significantly higher in the NCCN-defined high-risk group (*p* = 0.044) and patients aged >70 years (*p* = 0.007). In the 19 high-risk cases, the acquisition of missense mutations marginally predicted short DFS (Figure 4C, *p* = 0.0506). Among all the 58 GISTs sequenced, 20 cases developed tumor relapse, including 5 and 15 cases with and without *HSD11B1* mutations, respectively. The mean postoperative DFS duration of mutated and non-mutated GIST cases was 9.7 ± 6.38 and 35.6 ± 32.25 months, respectively (*p* = 0.033). However, *HSD11B1* mutations were not significantly related to the occurrence of CNG in a positive or inverse manner (*p* = 0.345), and there was no association between *HSD11B1* mutations and immunoexpression H-score (*p* = 0.851). 

### 3.6. Pro-Proliferative Role of HSD11B1 In Vitro

**Figure 5:**
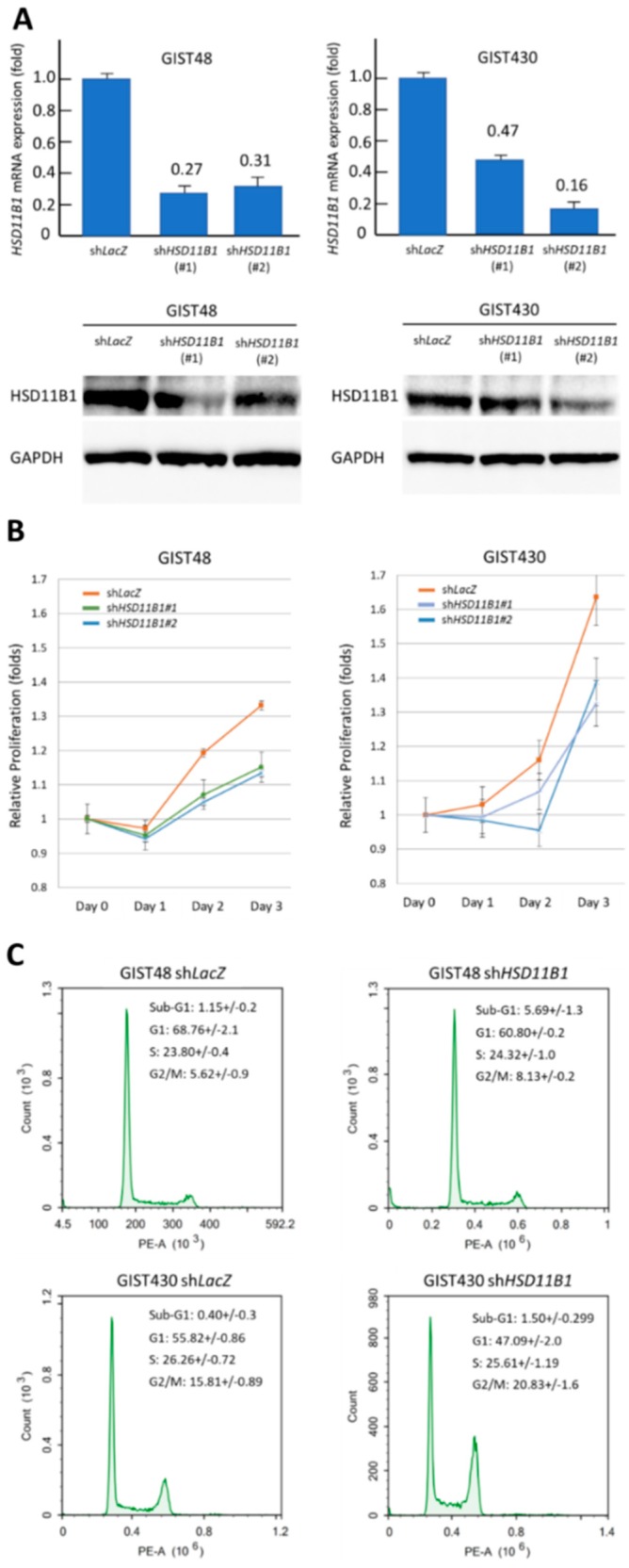
Validation of pro-proliferative oncogenic property of hydroxysteroid 11-beta dehydrogenase-1 (HSD11B1) in the gastrointestinal stromal tumor (GIST) cell models. (**A**) Quantitative RT-PCR (upper) and western blotting (lower) assays validated that stable transduction with either s*hHSD11B1 #1* or s*hHSD11B1 #2* effectively decreased *HSD11B1* mRNA and protein expression levels compared to stable transduction with *shLacZ* in the HSD11B1-expressing GIST48 (left) and GIST430 (right) cell lines; (**B**) In bromodeoxyuridine BrdU assay, the proliferation rate of GIST48 and GIST430 cell lines significantly decreased from 48 h onwards after transduction with s*hHSD11B1#1* or s*hHSD11B1#2*; (**C**) In flow cytometry-based cell cycle kinetic analysis, significantly increased percentages of tumor cells gated at the G_2_/M phase in GIST48 (upper) and GIST430 (lower) cell lines after s*hHSD11B1* transduction were observed. PE-A, phycoerythrin area.

As both *HSD11B1* CNG and high immunoexpression exhibited a strong prognostic negative effect, RNA interference was applied in GIST48 and GIST 430 cell lines to gain insight into the potential oncogenic role of HSD11B1. Each cell line was stably transduced with either of the two *shHSD11B1* clones or *shLacZ* control and validated by performing qPCR and western blotting assay (Figure 5A). In BrdU assay, both *shHSD11B1* clones significantly decreased the proliferation rates of GIST48 and GIST430 cells from 48 h after transduction compared to shLacZ control (Figure 5B), validating the in vitro pro-proliferative function of HSD11B1 in GISTs. In flow cytometric analysis, stable *HSD11B1* silencing in both GIST cell lines significantly increased the percentage of tumor cells in the G2/M phase (Figure 5C), implying that HSD11B1 might exert its pro-proliferative effect partially by promoting progression through the G2/M checkpoint. However, stable *HSD11B1* knockdown in both the cell lines did not significantly alter the cell migration and invasion potentials (Supplementary Figure S2). 

## 4. Discussion 

Metabolic reprogramming represents an essential difference between non-transformed and neoplastic cells [2]. In this study, we detected CNG and somatic mutations in *HSD11B1* gene, which regulates steroid and lipid metabolism [16] with each aberration exhibiting the same prevalence rate of 17% in primary imatinib-naïve GISTs. Contributing to the pro-proliferative phenotype, high HSD11B1 expression was also strongly associated with *HSD11B1* CNG that conferred a strong negative prognostic effect independent of the influence of high risk levels in GISTs. Although the missense mutations were not mutually exclusive or interlinked to the *HSD11B1* copy-number status, missense mutations trended toward a potentially earlier disease relapse. 

The protein encoded by *HSD11B1* forms a dimeric enzyme in the endoplasmic reticulum to catalyze the bidirectional interconversion between active cortisol and inactive cortisone [15,16]. Depending on the relative ratio of NADP to NADPH, this enzyme effectively orchestrates the homeostasis of glucocorticoid metabolism [15,16]. During the elevation of reverse cortisone oxo-reductase activity of HSD11B1, the excessive and chronically sustained cortisol might promote adipocyte differentiation and inhibit pre-adipocyte proliferation, hence causing metabolic syndrome and dyslipidemia [15,16,17]. In patients with cortisone-reductase deficiency, the mutations in *HSD11B1* or hexose-6-phosphate dehydrogenase (*H6PD*) that encodes an enzyme furnishing cofactors for the reaction might abrogate cortisol generation and consequently stimulate adrenal hyperandrogenism mediated by adrenocorticotropic hormone [25,26]. Compared with HSD11B2 isoform, the role of HSD11B1 in tumor biology remains elusive although an increased *HSD11B1* mRNA expression was reported in adrenal cortical neoplasms and colorectal cancers [27,28]. However, these limited studies regarding HSD11B1 lacked extensive elucidation of its clinical relevance, oncogenic functions, and potential molecular regulatory mechanisms. In this study, we provided functional evidence that stable *shHSD11B1* transduction significantly decreased the cell proliferation rate with concomitant cell cycle arrest at the G_2_/M checkpoint in two GIST cell lines that express wild-type HSD11B1. In the committed pre-adipocytes, a switch to the oxo-reductase activity of HSD11B1 is known to increase the cortisol generation that enhances the differentiation of these cells into mature fat cells instead of promoting proliferation [29]. In the cellular context of GIST, it is plausible that the increased HSD11B1 level renders imbalanced predominance toward dehydrogenation and in turn favors cell proliferation. 

Metabolic demands for rapid proliferation in common carcinomas were recently reported to exert a selection force that underpins conserved amplified DNA regions, which not only span classical oncogenes but also harbor a multitude of cancer-associated metabolic genes, such as glycolytic genes [30]. Additionally, cumulative DNA copy number alterations attract increasing attention toward their dosage effects on the regulation of lipid metabolic gene expression [31]. As a common event in breast carcinomas, the chromosome 8p deletion might allow tumor growth under stress conditions by reprogramming the fatty acid and ceramide metabolism to promote tumor progression and drug resistance [31]. These lines of evidence emphasize a notion that copy-number alterations involving metabolic gene loci might dictate the metabolic deregulation to aggravate tumor behavior. Interestingly, a genome-wide SNP array study reported that 10.3% of GISTs exhibited recurrent chromosomal 1q gain that spanned a region of 232.4 Mb in length and harbored the *HSD11B1* locus at 1q32.2 [32]. 

*HSD11B1* CNG was not formally documented as a mechanism underlying its overexpression in oncogenesis. However, according to the provisional data catalogued in the cBioPortal platform for cancer genomics [33], *HSD11B1* amplification might occur in varying but significant proportions of common cancers, such as prostate (13.6–26.2%), breast (9.5–17.6%), and renal cell (9.1%) carcinomas. By performing FISH assay, we substantiated the clinical relevance of increased dosage effect of *HSD11B1* with polysomy and amplification that were collectively detected in 17.4% of GIST cases. Our findings also underscored the presumable conversion of CNG into the upregulated mRNA and overexpressed protein of HSD11B1 that helped to identify aggressive GISTs as inferred from the strong correlations among these alterations in gene, mRNA, and protein. Compared with the non-neoplastic tissues, the whole group of GISTs exhibited a significantly higher *HSD11B1* mRNA level, which mainly resulted from the variation between non-high-risk and high-risk cases and suggested its occurrence as a non-early event. At the genetic and protein levels, *HSD11B1* CNG and elevated HSD11B1 expression highly characterized the GISTs that exhibit epithelioid histology, increased tumor size and mitosis, and high-risk levels. Therefore, the oncogenic potential of HSD11B1 is critically determined by CNG as a manifestation of increased genetic instability in GISTs. However, the modest association between *HSD11B1* CNG and unfavorable *KIT/PDGFRA/BRAF* genotypes was intriguing but still unaccountable. 

Unlike amplification, polysomy is not clearly established in human cancers regarding its clinical and biological relevance. *HSD11B1* polysomy strongly predicted remarkably short DFS compared to normal gene copies; however, it exhibited no significant difference from *HSD11B1* amplification in terms of tumor relapses. In addition, elevated HSD11B1 immunoexpression and *HSD11B1* CNG were both strongly predictive of short univariate DFS that resonates with the growth advantages contributed by HSD11B1 to exasperate the progression of GISTs. Notably, approximately two-thirds of high HSD11B1-expressing GISTs were normal in gene copy-number, conceivably indicating the existence of alternative regulatory mechanism(s) of *HSD11B1* at the mRNA and/or protein level. Although HSD11B1 overexpression was univariately effective to predict short DFS for all the GISTs in the TMA cohort and those with normal gene copies, its prognostic impact was not as prominent as that of CNG. Actually, CNG distinguished worse outcomes in GISTs with overexpressed HSD11B1 and surpassed the significance of high HSD11B1 expression in the multivariate regression model that incorporated the risk levels of either NCCN or NIH scheme. 

Deregulated metabolic enzymes are known to be directly involved in tumorigenesis through the gene mutations [1,4]. For example, the loss-of-function SDH complex mutations are considered to play a critical role in tumor initiation of a minor subset of GISTs that exhibit distinct clinicopathological features from GISTs with KIT- or PDGFRA-mutations [4,5,6]. In our sequencing studies, solely 17.2% of GISTs harbored non-synonymous missense *HSD11B1* mutations, and this low prevalence indicated their role as secondary aberrations in the cancer hallmark of metabolic deregulation among the GISTs that mostly manifested mutated-*KIT* or -*PDGFRA*. However, *HSD11B1* mutations are probably clinically and biologically relevant based on the following reasons. Firstly, the missense mutations in the exon-7 and -6 that affected the homodimer interface accounted for the vast majority of detected mutations and only one case exhibited an extra exon-3 mutation (p.M50V) apart from the frequently mutated exon-7 (p.E244K). Secondly, 7 of 12 mutated codons that were predicted using the PolyPhen-2 platform [34] probably damage the HSD11B1 function and are mapped to the exon-7 or exon-6. This manifestation is similar to cortisone-reductase deficiency characterized by mutations that interfere with the dimer assembly and abolish the reverse oxo-reductase activity [26]. Thirdly, our correlation analysis showed the significant prevalence of *HSD11B1* mutations in GISTs that belonged to high-risk category; *HSD11B1* mutations were also marginally and significantly associated with short DFS in high-risk GISTs and GISTs with tumor relapses, respectively. Notably, the occurrence of *HSD11B1* mutations and CNG were neither mutually exclusive nor significantly concomitant, and the lack of association between *HSD11B1* mutation and immunoexpression suggested that these mutations might solely affect the enzymatic function but not the expression level. 

In conclusion, we have characterized that HSD11B1 exhibits oncogenic potential in primary imatinib-naïve GISTs that is driven by CNG and/or missense mutations. *HSD11B1* CNG represents a critical mechanism that converts the increased gene dosage into upregulated mRNA and overexpressed protein. These aberrations might lead to aggressive GISTs with worse outcomes partially through the validated pro-proliferative function. However, the frequencies of CNG and protein overexpression of HSD11B1 were discrepant and not equivalent in their prognostic impact, with CNG being a stronger independent negative predictor. Predominantly affecting exon-7 or exon-6, missense *HSD11B1* mutations were present in 17% of GISTs and preferentially represented in old patients, high-risk cases, and probably in the subset prone to early relapse. Hence, our findings provide a rationale for future investigation on the utility of *HSD11B1* CNG and HSD11B1 immunoexpression as prognostic adjuncts in primary imatinib-naïve GISTs and shed light on the potential opportunity of inhibiting HSD11B1 as an alternative targeted therapeutic strategy in imatinib-resistant GISTs.

## Figures and Tables

**Table 1:** Associations of HSD11B1 expression and *HSD11B1* gene copy number with various clinicopathological parameters in 350 GIST patients.

Parameters	HSD11B1 Expression		*p*-Value	*HSD11B1* Copy Number		*p*-Value
	Low	High		Normal	Gain	
Sex			0.748			0.966
Male	85	88		143	30	
Female	90	87		146	31	
Age (years) ^&^	59.99 ± 12.867	59.74 ± 12.693	0.992	60.05 ± 12.974	59.03 ± 11/778	0.579
Location			<0.001 *			0.424
Gastric	122	89		177	34	
Non-gastric	53	86		112	27	
Histologic type			0.001 *			0.002 *
Spindle	146	120		229	37	
Epithelioid & mixed	29	55		60	24	
Tumor size (cm) ^&^	5.069 ± 3.007	7.741 ± 4.806	<0.001 *	5.870 ± 3.751	8.941 ± 5.305	<0.001 *
Mitotic count (50HPFs) ^&^	5.36 ± 17.191	13.10 ± 27.827	<0.001 *	6.74 ± 19.687	21.02 ± 34.040	<0.001 *
NIH risk level			<0.001 *			<0.001 *
Low/very low	88	39		118	9	
Intermediate	58	52		95	15	
High	29	84		76	37	
NCCN risk level			<0.001 *			<0.001 *
None/very low	69	19		85	3	
Low	60	40		87	13	
Moderate	33	32		54	11	
High	13	84		63	34	
Mutation type			0.452			0.015 *
Favorable type	48	58		90	16	
Unfavorable type	43	64		76	31	
HSD11B1 expression						<0.001 *
Normal	-	-		171	4 (polysomy)	
Gain	-	-		118	57 (polysomy, 4; amplification, 42)	

* Statistically significant; ^&^ Wilcoxon rank-sum test, HSD11B1, hydroxysteroid 11-beta dehydrogenase-1, NIH, National Institute of Health, NCCN, National Comprehensive Cancer Network. HPFs, high power fields.

**Table 2:** Univariate and multivariate analyses for disease-free survival according to gene and expression statuses of HSD11B1, NCCN risk levels, and other prognostic factors in 350 GISTs.

Parameters	Univariate Analysis			Multivariate Analysis		
	No. Case	No. Event	*p*-Value	HR	95% CI	*p*-Value
Sex			0.4667			
Male	177	43				
Female	173	44				
Age (years)			0.0584			
<70	259	59				
≥70	91	28				
Location			0.0023 *			0.796
Gastric	211	40		1	-	
Non-gastric	139	47		1.068	0.649–1.757	
Histologic type			<0.0001 *			0.013 *
Spindle	266	51		1	-	
Mixed/epithelioid	84	36		1.887	1.142–3.117	
Tumor size (cm) ^#^			<0.0001 *			
≤5 cm	161	16				
>5; ≤10 cm	131	38				
>10 cm	58	33				
Mitotic count (50HPFs) ^#^			<0.0001 *			
0–5	249	33				
6–10	43	14				
>10	58	40				
NCCN guideline			<0.0001 *			<0.001 *
None/very low	88	3		1	-	
Low	100	10		2.284	0.468–11.146	
Moderate	65	15		2.252	0.456–11.114	
High	97	59		8.344	1.793–38.826	
Mutation type			0.0005 *			0.418
Favorable type	106	22		1	-	
Unfavorable type	107	45		1.256	0.724–2.117	
HSD11B1 expression ^#^			<0.0001 *			0.354
Low expression	175	16		1	-	
High expression	175	71		1.402	0.687–2.860	
*HSD11B1* copy number			<0.0001 *			<0.001 *
Normal	289	45		1	-	
Gain	61	42		3.124	1.839–5.307	

**^#^** Tumor size and mitotic activity were not introduced in multivariate analysis, since these two parameters were component factors of NCCN risk scheme; * Statistically significant; HR, hazard ratio; CI, confidence intervals; HSD11B1, hydroxysteroid 11-beta dehydrogenase-1; GISTs, gastrointestinal stromal tumors; NCCN, National Comprehensive Cancer Network; HPFs, high power fields.

## Supplementary Materials

The following are available online at http://www.mdpi.com/2077-0383/7/11/408/s1, Supplementary methods: Transduction of *shHSD11B1*; Quantification of *HSD11B1* mRNA level in vitro; Western blotting analysis; Bromodeoxyuridine (BrdU) assay; Flow cytometry-based cell cycle kinetic assay; Cell migration and invasion assays, Figure S1: Chromatographs of Sanger sequencing for remaining six cases harboring *HSD11B1* mutations not illustrated in the Figure 4, Figure S2: The histograms of both GIST430 and GIST48 cell lines illustrating no statistical difference in the cell migratory (upper) and invasive (lower) capacities between *shLacZ* and *shHSD11B1* transduction conditions, Table S1: The clincopathological and mutational characters of 58 gastrointestinal tumors sequenced for *HSD11B1* gene, Table S2: Primer sequences, thermal conditions, and amplicon sizes of PCR-based *HSD11B1* mutation analysis, Table S3: Summary of differentially expressed genes associated with lipid metabolic process (GO:0006629) and exhibiting significant positive correlations with high-risk and development of metastasis in the transcriptome of GIST (GSE8167), Table S4: Univariate and multivariate analyses for disease-free survival according to gene and expression statuses of HSD11B1, NIH criteria, and other prognostic factors in 350 GISTs.
