# Supplementary material for: Hydroxysteroid 11-Beta Dehydrogenase 1 Overexpression with Copy-Number Gain and Missense Mutations in Primary Gastrointestinal Stromal Tumors
Source: J Clin Med. 2018 Nov 1;7(11):408. doi: 10.3390/jcm7110408 (PMC6262574; doi:10.3390/jcm7110408)
Supplement: Supplementary file 1 [file jcm-07-00408-s001.pdf]

Supplmetary Documents

1. Supplementary Methods

1.1. Transduction of shHSD11B1

The lentiviral vectors purchased from the Taiwan National RNAi Core Facility were pLKO.1-*shHSD11B1* (TRCN0000028045, target sequence 5′-GCTCCAAGGAAAGAAAGTGAT-3′ designated as *shHSD11B1*#1; TRCN0000028065, target sequence 5′-CGAGCTATAATATGGACAGAT-3′ designated as *shHSD11B1*#2). Their efficiency in stable knockdown of hydroxysteroid 11-beta dehydrogenase-1 (HSD11B1) was confirmed by quantitative real time polymerase chain reaction as well as western blotting assays as described below. pLKO.1-*shLacZ* (TRCN0000072223: 5′-TGTTCGCATTATCCGAACCAT-3′) vector was used as the nom-targeting control. Lentiviral vectors were produced by transfecting HEK293 cells with the aforementioned vectors using Lipofectamine 2000 (Invitrogen, Carlsbad, CA, USA). To perform viral infection, 3×10^6 cells were incubated with 8 mL lentiviral solution in the presence of polybrene, followed by selection using puromycin to identify the stable clones of lentivirus-transduced cells.

1.2. Quantification of HSD11B1 mRNA Level in Vitro

RNeasy Mini kit (Qiagen, Valencia, CA, USA) was used to extract total RNA from GIST cells with stable HSD11B1 knockdown, overexpression, or corresponding control vectors. RNA was reverse-transcribed using SuperScript™ III First-Strand Synthesis System (Invitrogen) according to the manufacturer's instructions. ABI StepOnePlus™ System was applied to perform qPCR and determine HSD11B1 mRNA expression level using pre-designed TaqMan assay reagents (Hs01547870_m1 for HSD11B1 and Hs01108291_mL for RNA polymerase polypeptide A [POLR2A]; Applied Biosystems, Foster City, CA, USA). The data were normalized using the expression of POLR2A housekeeping transcript. The relative fold change in the expression level of HSD11B1 mRNA was assessed by 2-ΔΔCp method.

1.3. Western Blotting Analysis

Western blotting assay was performed to evaluate the endogenous HSD11B1 expression and the efficiency of HSD11B1 knockdown in GIST cell lines. Total cell lysates that contain 25 μg protein were resolved by using 4–12% gradient NuPAGE gels (Invitrogen), electrophoretically transferred onto PVDF membranes (Amersham, Little Chalfont, UK), and probed with antibodies against HSD11B1 (Clone EPR9407(2), 1:10000, Abcam, London, UK) or GAPDH (1:3000, Chemicon, Tokyo, Japan). After incubation with appropriate secondary antibody, protein bands were visualized using chemiluminescence system (Amersham).

1.4. Bromodeoxyuridine (BrdU) Assay

DNA synthesis was assessed using an enzyme-linked immunosorbent assay (ELISA)-based colorimetric BrdU assay (Roche Diagnostics, Risch-Rotkreuz, Switzerland) in GIST cells transduced with *shHSD11B1* or *shLacZ* control. The absorbance of samples was measured using an ELISA reader (Promega, Madison, WI, USA) at 450 nm with 690 nm as reference.

1.5. Flow Cytometry-Based Cell Cycle Kinetic Assay

Stable pools of GIST cells transduced with *shHSD11B1* or *shLacZ* were pelleted, fixed overnight in cold ethanol, washed in PBS that contains 10 mg/mL RNase, and labeled with propidium iodide (PI; 0.05 mg/mL). The cell cycle kinetic profile was analyzed by using FACScan flow cytometer (BD Biosciences, Franklin Lakes, NJ, USA) and WinMDI2.9 software to determine the percentage of cells in each phase of the cell cycle. A minimum of $>10^4$ cells were sorted after gating out the fixation artifacts and cell debris.

1.6. Cell Migration and Invasion Assays

The analysis of migration and invasion potential of GIST cells was performed using 24-well cell migration and invasion assay kits (Millipore) by following the manufacturer's protocol.

Table S1. The clincopathological and mutational characters of 58 gastrointestinal tumors sequenced for *HSD11B1* gene.

Case No.	Age/Sex	Risk Levels by NCCN Guideline	Location	Gene Copy	Exon 2	Exon 3	Exon 4	Exon 6	Exon 7
1	54/M	High	Stomach	Gain	wt	wt	wt	wt	p.Y284C (c.851A > G), p.P271P(c.813A > C)
2	71/F	High	Stomach	Polysomy	wt	wt	wt	wt	p.G248R [#] (c.742G > A)
3	78/M	High	Stomach	Normal	wt	p.A55A (c.165G > A)	wt	wt	p.E239K (c.715G > A), p.C241Y (c.722G > A), p.S281L (c.842C > T)
4	71/F	High	Stomach	Normal	wt	wt	wt	wt	p.H232Y (c.694C > T)
5	80/F	High	Small intestine	Normal	wt	wt	wt	p.R205R (c.615G > A)	p.R252H (c.755G > A)
6	59/F	High	Small intestine	Normal	wt	wt	wt	wt	p.A223A (c.669C > T), p.H232Y (c.694C > T), p.E244E (c.732G > A)
7	75/M	High	Stomach	Gain	wt	wt	wt	wt	wt
8	75/F	High	Stomach	Normal	wt	wt	wt	wt	wt
9	60/M	High	Stomach	Gain	wt	wt	wt	wt	wt
10	32/F	High	Stomach	Gain	wt	wt	wt	wt	wt
11	59/F	High	Stomach	Gain	wt	wt	wt	wt	wt
12	36/F	High	Stomach	Normal	wt	wt	wt	wt	wt
13	71/F	High	Small intestine	Normal	wt	wt	wt	wt	wt
14	58/F	High	Small intestine	Normal	wt	wt	wt	wt	wt
15	72/M	High	Small intestine	Normal	wt	wt	wt	wt	wt
16	66/M	High	Small intestine	Normal	wt	wt	wt	wt	wt
17	52/F	High	Small intestine	Normal	wt	wt	wt	wt	wt
18	52/F	High	Small intestine	Normal	wt	wt	wt	wt	wt
19	57/M	High	Small intestine	Gain	wt	wt	wt	wt	wt
20	50/F	Moderate	Stomach	Normal	wt	wt	wt	wt	p.L267L (c.801G > A), p.M286I (c.858G > A)
21	68/M	Moderate	Small intestine	Normal	wt	wt	wt	wt	p.V256V (c.768G > A)
22	73/M	Moderate	Stomach	Gain	wt	wt	wt	wt	wt
23	67/F	Moderate	Stomach	Normal	wt	wt	wt	wt	wt
24	46/M	Moderate	Stomach	Normal	wt	wt	wt	wt	wt
25	67/M	Moderate	Stomach	Normal	wt	wt	wt	wt	wt
26	64/F	Moderate	Stomach	Gain	wt	wt	wt	wt	wt
27	59/F	Moderate	Stomach	Normal	wt	wt	wt	wt	wt
28	71/F	Moderate	Stomach	Gain	wt	wt	wt	wt	wt
29	66/F	Moderate	Stomach	Normal	wt	wt	wt	wt	wt
30	63/F	Moderate	Stomach	Normal	wt	wt	wt	wt	wt
31	41/F	Moderate	Small intestine	Gain	wt	wt	wt	wt	wt
32	65/F	Moderate	Small intestine	Normal	wt	wt	wt	wt	wt
33	57/M	Moderate	Small intestine	Normal	wt	wt	wt	wt	wt
34	38/M	Moderate	Small intestine	Normal	wt	wt	wt	wt	wt

35	34/M	Moderate	Small intestine	Normal	wt	wt	wt	wt	wt
36	66/F	Moderate	Small intestine	Normal	wt	wt	wt	wt	wt
37	64/F	Moderate	Small intestine	Gain	wt	wt	wt	wt	wt
38	40/F	Moderate	Small intestine	Normal	wt	wt	wt	wt	wt
39	53/F	Moderate	Small intestine	Normal	wt	wt	wt	wt	wt
40	75/M	Low	Small intestine	Normal	wt	wt	wt	p.F193S (c.578T > C)	wt
41	24/F	Low	Small intestine	Normal	wt	p.E30E (c.90G > A)	wt	wt	p.S283N (c.848G > A)
42	60/F	Low	Stomach	Normal	wt	wt	wt	wt	wt
43	56/M	Low	Stomach	Gain	wt	wt	wt	wt	wt
44	56/F	Low	Stomach	Gain	wt	wt	wt	wt	wt
45	70/M	Low	Stomach	Gain	wt	wt	wt	wt	wt
46	57/M	Low	Stomach	Normal	wt	wt	wt	wt	wt
47	53/F	Low	Stomach	Normal	wt	wt	wt	wt	wt
48	62/F	Low	Stomach	Normal	wt	wt	wt	wt	wt
49	61/M	Low	Small intestine	Normal	wt	wt	wt	wt	wt
50	37/M	Low	Small intestine	Normal	wt	wt	wt	wt	wt
51	39/M	Low	Small intestine	Normal	wt	wt	wt	wt	wt
52	36/M	Very Low/Low	Small intestine	Gain	wt	wt	wt	wt	wt
53	34/M	Very Low/Low	Small intestine	Gain	wt	wt	wt	wt	wt
54	70/M	Very Low/Low	Small intestine	Gain	wt	wt	wt	wt	wt
55	84/F	Very Low	Small intestine	Normal	p.L9L (c.27C > T)	p.M50V (c.148A > G)	wt	wt	p.E244K (c.730G > A)
56	75/F	Very Low	Stomach	Normal	wt	wt	wt	wt	wt
57	40/M	Very Low	Stomach	Normal	wt	wt	wt	wt	wt
58	61/F	Very Low	Stomach	Normal	wt	wt	wt	wt	wt

To contrast with synonymous silent mutations, the non-synonymous missense mutations are expressed in bold fonts. HSD11B1, hydroxysteroid 11-beta dehydrogenase-1; NCCN, National Comprehensive Cancer Network.

Table S2. Primer sequences, thermal conditions, and amplicon sizes of PCR-based *HSD11B1* mutation analysis.

Exon	Forward	Reverse	Annealing Temperature	Amplicon Size
Exon 2	GCTGCCTGCTTAGGAGGTT	CTATTCCTCCAAAATGAGACGCA	58°C	205 bp
Exon 3-a *	TGCCTATATCCAGAGAGGGAGAA	CATCTGAGCATGTGACGGTA	58°C	256 bp
Exon 3-b *	ATTTTGCTGCCAACTTGGGT	CATCTGAGCATGTGACGGTA	58°C	230 bp
Exon 4	CTAAGACTGATGCCATTTCTGCT	GTTGATACCTTTCCCATTCTCCT	58°C	234 bp
Exon 6	AGGTGAAATGGGCAGCCTTA	ACAAAGTATTGACCTTACCTGTG TC	58°C	211 bp
Exon 7	ACCCTACTCTTCCCTTGTCA	AGTCCCAAAATCCCTCAGCA	58°C	287 bp

Thermal conditions: For formalin-fixed, paraffin-embedded tissues, genomic DNA from each GIST sample was subjected to 40 cycles of PCR in a final reaction volume of 25μL, which contained 0.2 μM of each oligonucleotide primer, 2.5U Platinum Taq DNA polymerase (Invitrogen, Carlsbad, CA, USA), 0.5 μL of dNTP mixture at 10 mM, 18 μL ddH_2O, 1.5 mM $MgCl_2$, and 2.5 μL of 10 × PCR buffer. PCR conditions were 95°C for 30 sec, 58°C (i.e., the same annealing temperature of each primer pair) for 30 sec, and 72°C for 45 sec. *, In general, the primer pair 3-a targeting HSD11B1 exon 3 with a larger amplicon size was first tested. In 4 cases, an alternative primer pair 3-b was then attempted when the initial primer pair 3-a failed to yield PCR

products. For cell line samples, the identical primer pairs and reagents were used, while the thermal protocol was reduced to 30 PCR cycles, with each starting with 95°C for 5 min, 58°C for 30 sec, and 72°C for 10 min as the final elongation step. HSD11B1, hydroxysteroid 11-beta dehydrogenase-1.

Table S3. Summary of differentially expressed genes associated with lipid metabolic process (GO: 0006629) and showed positive associations to high-risk and development of metastasis in the transcriptome of the gastrointestinal stromal tumor (GIST) (GSE8167).

**Probe**	**High vs. Non-High Risk**		**Meta. vs. Non-Meta.**		**Gene Symbol**	**Gene Name**	**Biological Process**	**Molecular Function**
	**Log Ratio**	***p*-Value**	**Log Ratio**	***p*-Value**				
203895_at	1.2642	<0.0001	1.0554	0.0011	*PLCB4*	phospholipase C; beta 4	intracellular signaling cascade, lipid catabolic process, lipid metabolic process, signal transduction	calcium ion binding, hydrolase activity, phosphoinositide phospholipase C activity, phospholipase C activity, protein binding, signal transducer activity
203896_s_at	1.1827	0.0001	0.9318	0.0071	*PLCB4*	phospholipase C; beta 4	intracellular signaling cascade, lipid catabolic process, lipid metabolic process, signal transduction	calcium ion binding, hydrolase activity, phosphoinositide phospholipase C activity, phospholipase C activity, protein binding, signal transducer activity
205404_at	2.7352	<0.0001	2.3984	0.0001	*HSD11B1*	hydroxysteroid (11-beta) dehydrogenase 1	lipid metabolic process, lung development, metabolic process, steroid metabolic process	11-beta-hydroxysteroid dehydrogenase (NADP+) activity, 11-beta-hydroxysteroid dehydrogenase activity, oxidoreductase activity
227038_at	0.9678	0.0031	1.0225	0.0045	*SGMS2*	sphingomyelin synthase 2	lipid metabolic process, sphingolipid metabolic process, sphingomyelin biosynthetic process	ceramide cholinephosphotransferase activity, kinase activity, sphingomyelin synthase activity, transferase activity
242963_at	0.523	<0.0001	0.618	<0.0001	*SGMS2*	sphingomyelin synthase 2	lipid metabolic process, sphingolipid metabolic process, sphingomyelin biosynthetic process	ceramide cholinephosphotransferase activity, kinase activity, sphingomyelin synthase activity, transferase activity
211026_s_at	1.3407	<0.0001	0.9133	0.0006	*MGLL*	monoglyceride lipase	aromatic compound metabolic process, inflammatory response, lipid metabolic process	acylglycerol lipase activity, catalytic activity, hydrolase activity, lysophospholipase activity
209121_x_at	0.907	<0.0001	0.7189	0.0035	*NR2F2*	nuclear receptor subfamily 2; group F; member 2	lipid metabolic process, regulation of transcription from RNA polymerase II promoter, regulation of transcription; DNA-dependent, signal transduction, transcription	DNA binding, ligand-dependent nuclear receptor activity, ligand-regulated transcription factor activity, metal ion binding, receptor activity, sequence-specific DNA binding, steroid hormone receptor activity, transcription corepressor activity, transcription factor activity, zinc ion binding
211708_s_at	0.2555	0.0012	0.2394	0.0064	*SCD*	stearoyl-CoA desaturase (delta-9-desaturase)	fatty acid biosynthetic process, lipid biosynthetic process, lipid metabolic process	iron ion binding, oxidoreductase activity, oxidoreductase activity; acting on paired donors; with oxidation of a pair of donors resulting in the reduction of molecular oxygen to two molecules of water, stearoyl-CoA 9-desaturase activity
212657_s_at	0.4939	0.0033	0.8933	<0.0001	*IL1RN*	interleukin 1 receptor antagonist	immune response, inflammatory	interleukin-1 receptor antagonist activity, interleukin-1 receptor

							response, insulin secretion, lipid metabolic process	binding, protein binding, receptor activity
208383_s_at	0.3154	0.0094	0.6032	<0.0001	*PCK1*	phosphoenolpyruvate carboxykinase 1 (soluble)	gluconeogenesis, glucose metabolic process, glycerol biosynthetic process from pyruvate, lipid metabolic process	GTP binding, carboxy-lyase activity, lyase activity, nucleotide binding, phosphoenolpyruvate carboxykinase (GTP) activity, phosphoenolpyruvate carboxykinase activity, purine nucleotide binding

Table S4. Univariate and multivariate analyses for disease-free survival according to gene and expression statuses of HSD11B1, NIH criteria, and other prognostic factors in 350 GISTs.

**Parameter**	**Univariate Analysis**			**Multivariate Analysis**		
	**No. Case**	**No. Event**	***p*-Value**	**HR**	**95% CI**	***p*-Value**
Sex			0.4667			
Male	177	43				
Female	173	44				
Age (years)			0.0584			
<70	259	59				
≥70	91	28				
Location			0.0023 *			0.081
Gastric	211	40		1	-	
Non-gastric	139	47		1.431	0.874–2.343	
Histologic type			<0.0001 *			0.031 *
Spindle	266	51		1	-	
Mixed/epithelioid	84	36		1.793	1.084–2.967	
Tumor size (cm) [#]			<0.0001 *			
≤5 cm	161	16				
>5; ≤10 cm	131	38				
>10 cm	58	33				
Mitotic count (50HPFs) [#]			<0.0001 *			
0–5	249	33				
6–10	43	14				
>10	58	40				
NIH consensus			<0.0001 *			<0.001 *
Very low/low	127	6		1	-	
Intermediate	110	17		1.069	0.367–3.113	
High	113	64		5.189	1.971–13.662	
Mutation type			0.0005 *			0.318
Favorable type	106	22		1	-	
Unfavorable type	107	45		1.784	1.058–3.009	
HSD11B1 expression			<0.0001 *			0.095
Low expression	175	16		1	-	
High expression	175	71		2.601	1.370–4.937	
HSD11B1 copy number			<0.0001 *			<0.001 *
Normal	289	45		1	-	
Gain	61	42		3.127	1.823–5.364	

[#], Tumor size and mitotic activity were not introduced in multivariate analysis, since these two parameters were component factors of NIH risk scheme; *, Statistically significant; HR, hazard ratio; CI, confidence intervals; GISTs, gastrointestinal stromal tumors; HSD11B1, hydroxysteroid 11-beta dehydrogenase-1; NIH, National Institute of Health; HPFs, high power fields.

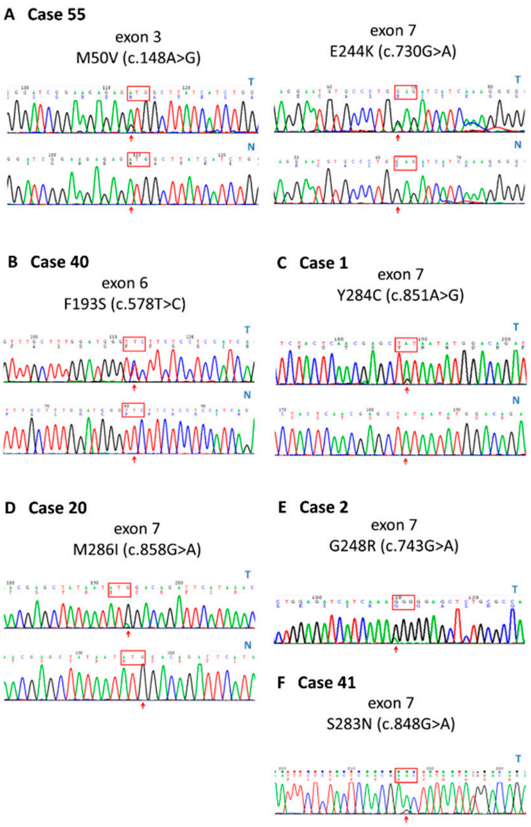

Figure S1. Chromatographs of Sanger sequencing for remaining six cases harboring *HSD11B1* mutations not illustrated in the Figure 4. Single or double non-synonymous *HSD11B1* mutations were also detected in the remaining 6 primary GISTs, including p.M50V in exon 3 and p.E244K in exon 7 **(A,** case 55**)**, p.F193S in exon 6 **(B,** case 40**)**, as well as p. Y284C **(C,** case 1**)**, p.M286I **(D,** case 7**),** p.G248R **(E,** case 2**)**, p.S283N **(F**, case 41**)** in exon 7. Of these, four cases with matched adjacent normal tissues (**A to D**) were sequenced in parallel for comparison. **T**: GIST tissue; **N**: normal tissue.

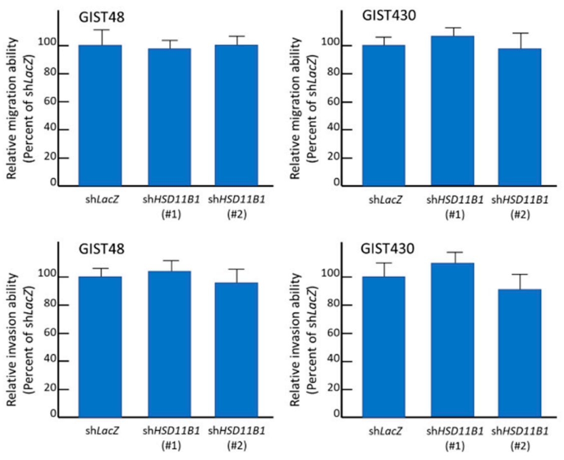

Figure S2. The histograms of both GIST430 and GIST48 cell lines illustrating no statistical difference in the cell migratory (upper) and invasive (lower) capacities between shLacZ and shHSD11B1 transduction conditions. GIST, the gastrointestinal stromal tumor.
